# Hypothalamus-pituitary-gonad axis transcriptome profiling for sex differentiation in *Acipenser sinensis*

**DOI:** 10.1038/s41597-019-0099-1

**Published:** 2019-06-13

**Authors:** Hejun Du, Jianbo Jian, Binzhong Wang, Xueqing Liu, Jianwei Chen, Kan Xiao, Jinquan Xia, Jing Yang, Yong Gao, Lei Chen

**Affiliations:** 1 Hubei Key Laboratory of Three Gorges Project for Conservation of Fishes, Yichang, Hubei 443100 China; 2 Chinese Sturgeon Research Institute, China Three Gorges Corporation, Yichang, Hubei 443100 China; 3 BGI genomics, BGI-Shenzhen, Shenzhen, Guangdong 518083 China; 4 BGI-Qingdao, BGI-Shenzhen, Qingdao, Shandong 266555 China

**Keywords:** RNA sequencing, Data publication and archiving, High-throughput screening

## Abstract

Chinese sturgeon (*Acipenser sinensis*), a critically endangered *Acipenseridae* family member, is one of the largest anadromous, native fish in China. Numerous research programmes and protection agencies have focused on breeding and preserving this endangered species. However, available information is limited on the different stages of sex development, especially on the reproductive regulation of the hypothalamus-pituitary-gonad (HPG) axis of *A. sinensis*. To unravel the mechanism of gene interactions during sex differentiation and gonad development of *A. sinensis*, we performed transcriptome sequencing using HPG samples from male and female *A. sinensis* in two developmental stages. In this study, 271.19 Gb high-quality transcriptome data were obtained from 45 samples belonging to 15 individuals (six in stage I, six males and three females in stage II). These transcriptomic data will help us understand the reproductive regulation of the HPG axis in the development stages of *A. sinensis* and provide important reference data for genomic and genetic studies in *A. sinensis* and related species.

## Abstract (summary)

**Table:** 

Design Type(s)	development or differentiation design • transcription profiling design • sequence assembly objective • sequence annotation objective
Measurement Type(s)	transcription profiling assay
Technology Type(s)	RNA sequencing
Factor Type(s)	sex • developmental stage
Sample Characteristic(s)	Acipenser sinensis • pituitary gland • Hypothalamus • Gonad

Machine-accessible metadata file describing the reported data (ISA-Tab format)

## Background & Summary

The order Acipenseriformes comprises 27 extant species of sturgeons, which are considered “living fossils” with a fossil record dating back to the upper Cretaceous^1,2^. In terms of scientific value, these chondrostean species preserve many transition traits between Chondrichthyes and Osteichthyes, occupying a vital evolutionary position. Sturgeons include three degrees of polyploidy, including ~120, ~240 and ~360 chromosomes, which might result from multiple and independent duplication events^3,4^. Sturgeons are important species for studying vertebrate evolution and whole genome duplication. Regarding economic value, black caviar is very popular in the European market, and sturgeon meat is also an important food resource^5^. Unfortunately, most sturgeons are currently endangered^6^.

Chinese sturgeon (*Acipenser sinensis*) is a large anadromous fish present in the main stream of the Yangtze River and East China Sea in China^5,7^. At present, Chinese sturgeon is critically endangered, which is mainly attributed to the following reasons: (i) asynchronous and delayed sexual maturity between male and female Chinese sturgeon (8–18 years for male and 14–26 years for female)^6^; and (ii) human activities, such as overfishing, damming, shipping and pollution^7^. The sex ratio (female:male) of wild Chinese sturgeon has gradually increased from 1.1:1 (1981–1983) to 5.86:1 (2003–2004)^8^. Therefore, artificial propagation and selective release are considered efficient approaches to recover the wild population size and adjust the sex ratio of the wild population of Chinese sturgeon. However, the sexes of *A. sinensis* cannot be distinguished due to a lack of secondary sexual characteristics and molecular markers of sex identification. Moreover, whether sex differentiation of *A. sinensis* is determined by genetics, the environment or a combination of both remains unclear.

Sturgeon genomes are complicated due to large genome sizes (1.6–9.32 pg/C), many chromosomes (consisting of macro- and micro-chromosomes) and controversial ploidy^4,9,10^. To date, no sturgeon reference genome is available. Transcriptome sequencing is very useful for identifying novel genes, checking gene activity, revealing genic functions, and exploring the molecular mechanisms of development and sex differentiation of sturgeon species^11,12^. Transcriptome analyses regarding sex determination and development of several sturgeons, including *A. naccarii*^13^, *A. schrenckii*^14–18^, *A. sinensis*^6,19,20^, *A. gueldenstaedtii*^21^, *A. fulvescens*^11^, *A. baerii*^22^, and *A. dabryanus*^23,24^, have been reported. Transcriptomes between male and female *A. sinensis* gonads were analysed in a previous study^6^ where some differential genes between the ovary and testis were predicted, and potential gametogenesis-related genes were screened out. However, identifying the sex determination mechanism of *A. sinensis* only by analysing genes in gonad tissues is not possible. The regulation of multiple tissues, signals and genes must be considered to unveil the mechanism of sex differentiation and discover sexual markers.

The reproductive process in vertebrates relies on multiple regulatory factors of the hypothalamus-pituitary-gonad (HPG) axis^25,26^. Gonadal differentiation, development, and maturation in fish are also regulated by many kinds of hormones, such as gonadotropin-releasing hormones, gonadotropin hormones, steroid hormones and other relative hormones, in the HPG axis^14,27,28^. Recent studies on the HPG axis of *A. schrenckii* focused only on the genes in the HPG axis from brain tissue by kisspeptin treatment^14^. However, obtaining transcriptome profiles of whole HPG tissues in sturgeon is necessary to better understand the reproductive mechanisms.

Here, the HPG axis transcriptomes of *A. sinensis* were sequenced using Illumina HiSeq 2000 and 4000 platforms. A total of 271.19 Gb filtered data from 45 samples belonging to 15 individuals (six individuals in stage I, six males and three females in stage II) were obtained, and 74.50% of 121,952 assembled unigenes were annotated to the NCBI non-redundant protein database (NR), Swiss-Prot, Kyoto Encyclopedia of Genes and Genomes (KEGG), Cluster of Orthologous Groups of proteins (COG), InterPro, Gene Ontology (GO) and NT databases. Additionally, 78,770 unigenes contained complete open reading frames (ORFs). A total of 51,570 simple sequence repeats (SSRs) were detected. We identified 1,531 to 34,439 differentially expressed genes (DEGs) in 18 pairwise comparisons of HPG samples of male and female *A. sinensis* in two stages. We reported the first integrated transcriptome data from HPG samples of male and female *A. sinensis* in stage I and II of gonadal development. These data offer valuable resources for research into reproductive regulation and the HPG axis interaction in *A. sinensis* and other sturgeons.

## Methods

### Ethics statement

The experiments were performed in accordance with the guidelines and regulations of the National Institute of Health Guide for the Care and Use of Laboratory Animals and were approved by the Institutional Review Board on Bioethics and Biosafety of the Chinese Sturgeon Research Institute.

### Sample preparation

Individuals of *A. sinensis* used here were selected from the artificial breeding population of the Chinese Sturgeon Research Institute, China Three Gorges Corporation (Yichang, China). The stages of gonadal development were classified following previously described methods^29^. The sex cannot be distinguished by a histochemical assay for gonads in stage I, while it can be easily distinguished in stage II in *A. sinensis*. Herein, 6 individuals (1 year old) in stage I and 9 individuals (6 males and 3 females over 4 years old) in stage II were selected. All experimental individuals were first euthanized with M222. Three kinds of tissues (hypothalamus (H), pituitary (P) and gonad (G)) were collected from each individual, flash frozen in liquid nitrogen and stored at −80 °C for RNA extraction. A total of 45 samples from 15 individuals (three tissues for each individual) were used for cDNA library construction.

### Illumina sequencing and data processing

Total RNA was extracted with TRIzol reagent (Invitrogen) following the manufacturer’s instructions. The RNA purity, integrity and concentration were calculated and checked by an Agilent 2100 Bioanalyzer (Agilent, Santa Clara, CA), and high-quality RNA (RNA Integrity Number > 7.0) was used for Illumina sequencing. Total RNA samples (5 μg) were then subjected to cDNA construction following Illumina truSeq stranded mRNA sample preparation protocol. Next, 45 libraries were sequenced using Illumina HiSeq^TM^ 2000/4000 with 2 × 90 or 100 bp paired-end sequencing. To obtain high-quality reads, the raw reads were filtered by SOAPnuke (v1.5.6)^30^ with the default parameters except “-l 20 -q 0.2 –M 3” according to the following criteria: (i) reads containing adaptors (adapters of more than 15 bases matched to reads with maximal 3-base mismatches allowed) were discarded, (ii) reads with a high proportion (> 5%) of unknown nucleotides (N) were removed, and (iii) reads with ≥ 20% bases Q ≤ 20 were removed. Here, the filtering parameters were stricter than the default parameters, and reads containing low quality bases or adapters were removed entirely, rather than being trimmed.

### Transcriptome assembly and annotation

All data obtained from the 45 libraries were assembled by the Trinity program (version: release-2013-08-14), including Inchworm, Chrysalis and Butterfly modules, with default parameters except “–path_reinforcement_distance 85 –min_kmer_cov 3”^31^. To minimize redundancy, we clustered transcripts using TGICL^32^ and the non-redundant sequences of >200 bp were retained. Finally, the longest sequence was preserved and designated as a unigene. Following the filtering methods of the unigene set of *Andrias davidianus*^33^, we used a strict pipeline to filter these sequences with some modification to reduce the background and assembly errors. There are some differences compared with the published filtered cut-offs^33^. Here, if the lengths of unigenes were in the range of 200–500 bp with the fragments per kilobase per million mapped fragments (FPKM) < 50 in just one sample or FPKM < 1 in ten samples, they were removed. Several different public protein databases were used to validate and annotate the assembled unigenes for assigning gene names, coding sequences (CDS) and predicting protein annotations. The sequence-based alignments were mapped against the NCBI NR protein database, Swiss-Prot protein database, KEGG and COG using the BLASTx algorithm^34,35^ with an E-value threshold of 1e^−5^. The priority order of NR, Swiss-Prot, KEGG, and COG was set. The unigenes that did not align to any of the above databases were predicted as ORFs using ESTScan software^36^. To further evaluate the different sample mapping rates and the quality of the assembled unigenes, we aligned all tissue samples with high-quality reads to the unigenes using SOAP2.21, allowing up to 5 base mismatches^37^.

To annotate and categorize *A. sinensis* unigene function, we conducted NR annotation, and GO analysis. The NR animal protein sequences came from dozens of species; based on NR annotation, unigenes were assigned to GO classes using BLAST2GO^38^. With WEGO software^39^, the assigned GO terms were summarized into the three main GO categories, including biological process, molecular function and cellular component. Overall, all the unigenes were assigned COG classifications based on all the unigenes blasted against the COG database. The COG-annotated putative proteins were classified into 25 functional categories. To assess the quality of the transcriptome gene set, we evaluated the completeness of coding gene set using Benchmarking Universal Single-Copy Orthologs (BUSCO)^40^.

### Differentially expressed gene analysis and RT-qPCR validation

To obtain the gene expression information, we mapped the high-quality reads of each sample to all transcripts using SOAP2 software^41^. After counting the number of mapped reads, estimated FPKM values were calculated through RSEM based on the mapping results. Principal component analysis (PCA) was performed for the 45 samples with the FPKM by the princomp function in the R package^42^. The 45 samples from the HPG in two stages were designed for 18 pairwise comparisons. The DEGs of these pairwise comparisons were evaluated by Noiseq software with probability no less than 0.8^43^. The GO annotation methods are similar to those mentioned above in unigene annotations. Compared to the whole genome background, the DEG pathway enrichment analysis was identified by a significantly enriched pathway. After multiple testing for the *p*-value, pathways with a *q*-value ≤ 0.05 were defined as significantly enriched pathways.

To validate the DEGs, we performed RT-qPCR to evaluate the sequencing and data analysis. Total RNA was extracted using TRIzol reagent (Ambion) according to the manufacturer’s protocol. Approximately 1 μg of RNA was converted to cDNA. Then, the cDNA templates were reverse-transcribed with a PrimeScript^TM^ RT reagent kit with gDNA Eraser (Perfect Real Time) (TakaRa, Dalian, China). RT-qPCRs were performed on a My IQTM colour Real-time PCR Detection System (Bio-Rad, USA). Using *β*-actin as a reference gene normalized by the median expression, the relative expression levels of 10 target sex-related genes were calculated by the 2^−ΔΔCt^ method^44^. RT-qPCR was performed on three biological replicates, and the data are presented as the mean ± SD. With Student's *t*-test, the difference was considered statistically significant at a *p*-value < 0.05.

### Microsatellite markers

SSRs were detected in unigenes by MIcroSAtellite (MISA; http://pgrc.ipk-gatersleben.de/misa, version 1.0) software. The following types of SSRs were detected: mono-, di-, tri-, tetra-, penta- and hexa-nucleotide repeats, as well as compound SSRs [the sequence including more than one type of repeat units, e.g., (GA)n(TC)m]. Here, the standards of the repeat unit numbers were set as follows: mono-10, dimer-6, trimer-5, tetramer-5, pentamer-5, and hexamer-5.

## Data Records

All raw reads of transcriptome sequences have been submitted to the NCBI Sequence Read Archive^45^. The assembled transcriptome data were deposited in NCBI’s GenBank^46^. The gene expression measurements were deposited in Gene Expression Omnibus (GEO)^47^. The transcriptome annotation of unigenes in the NR, NT, SwissProt, KEGG, COG, InterPro and GO database, differentially expressed genes in different comparisons, RT-qPCR test results and statistic of SSR motifs of the *Acipenser sinensis unigenes* can be found in our Figshare records^48^.

## Technical Validation

**Fig. 1:**
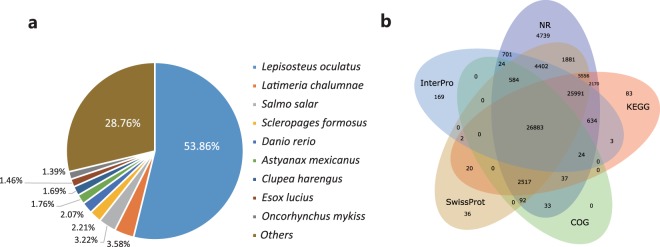
Characteristics of *Acipenser sinensis* unigenes with a BLASTx search against databases. (**a**) Species distribution of the best BLASTx matches for each unigene. (**b**) The annotation of the *Acipenser sinensi*s unigenes in the NR, InterPro, Swiss-Prot, COG and KEGG databases.

A total of 3,255.19 M raw reads were generated by Illumina HiSeq 2000 and 4000 platforms. After filtering raw reads, 2,967.34 M high-quality reads (271.19 Gb high-quality data) were used for the transcriptome assembly, and all 45 samples were used for differential expression analysis. Sample sequencing statistics and high-quality data information are presented in Table 1. The statistics of the assembled unigene set of the 45 samples are listed in Online-only Table 1; the total number ranged from 40,374 to 155,858. The mean length and N50 length of the 121,952 unigenes were 1,384 bp and 2,208 bp, respectively. To annotate and categorize *A. sinensis* unigene function, we conducted NR annotation and GO analysis. A total of 76,268 unigenes matched the NR animal species dataset with BLASTx. Then, 53.86% of the unigenes were most similar to the genes of *Lepisosteus oculatus*, followed by *Latimeria chalumnae* (3.58%), *Salmo salar* (3.22%), *Scleropages formosus* (2.21%), *Danio rerio* (2.07%), *Astyanax mexicanus* (1.76%) and *Clupea harengus* (1.69%) (Fig. 1a).

**Table 1:** Raw data, clean data, quality and GC content of 45 transcriptomes from the *Acipenser sinensis* hypothalamus-pituitary-gonad (HPG) axis in two sex development stages.

Sample Classification	Samples	Total Raw Reads	Total Clean Reads	Total Clean Nucleotides (nt)	Q20 (Clean data)	GC Content (Clean data)
Individuals of stage I (1 year old)	Asia1h	72,561,602	67,120,606	6,040,854,540	97.81%	46.06%
	Asia1p	70,982,504	66,170,244	5,955,321,960	97.75%	46.94%
	Asia1s	52,674,760	49,336,672	4,440,300,480	98.84%	49.79%
	Asia2h	69,592,162	64,500,510	5,805,045,900	97.83%	46.02%
	Asia2p	73,903,484	68,139,236	6,132,531,240	97.71%	46.86%
	Asia2s	67,369,164	63,892,914	5,750,362,260	98.88%	47.81%
	Asia3h	68,160,832	64,051,270	5,764,614,300	98.47%	47.22%
	Asia3p	69,409,406	63,942,236	5,754,801,240	97.63%	46.95%
	Asia3s	69,540,028	66,011,416	5,941,027,440	98.93%	48.42%
	Asia4h	69,525,450	64,765,390	5,828,885,100	98.44%	46.80%
	Asia4p	72,361,440	66,537,616	5,988,385,440	97.66%	46.70%
	Asia4s	69,299,166	66,394,148	5,975,473,320	98.93%	49.77%
	Asia5h	74,425,026	68,937,678	6,204,391,020	98.42%	47.79%
	Asia5p	73,305,770	67,419,728	6,067,775,520	97.69%	47.33%
	Asia5s	71,279,266	65,365,328	5,882,879,520	97.60%	45.52%
	Asia6h	74,365,432	69,060,388	6,215,434,920	98.39%	47.92%
	Asia6p	72,644,928	67,460,244	6,071,421,960	97.89%	46.33%
	Asia6s	73,273,656	66,293,454	5,966,410,860	97.42%	45.54%
Males of stage II (>4 years old)	Asib1Mh	72,188,720	63,966,898	5,757,020,820	97.07%	47.16%
	Asib1Mp	75,207,960	66,281,450	5,965,330,500	96.92%	49.41%
	Asib1Ms	72,592,714	64,475,040	5,802,753,600	97.03%	47.83%
	Asib2Mh	74,034,946	64,347,466	5,791,271,940	96.39%	47.05%
	Asib2Mp	75,130,342	66,688,110	6,001,929,900	97.03%	48.55%
	Asib2Ms	78,521,204	68,848,698	6,196,382,820	96.44%	47.63%
	Asib3Mh	73,725,658	64,308,048	5,787,724,320	96.39%	47.39%
	Asib3Mp	76,342,778	66,185,388	5,956,684,920	96.25%	49.09%
	Asib3Ms	72,932,412	64,883,560	5,839,520,400	97.28%	48.45%
	Asib4Mh	74,922,504	65,787,098	5,920,838,820	97.10%	47.83%
	Asib4Mp	72,112,948	64,477,384	5,802,964,560	97.31%	48.73%
	Asib4Ms	74,155,504	66,560,382	5,990,434,380	97.41%	47.62%
	Asib5Mh	74,257,722	65,455,208	5,890,968,720	97.30%	47.64%
	Asib5Mp	72,514,462	64,153,698	5,773,832,820	97.16%	48.83%
	Asib5Ms	72,364,498	64,203,156	5,778,284,040	97.20%	48.38%
	Asib6Mh	77,055,568	69,113,198	6,220,187,820	98.16%	47.90%
	Asib6Mp	71,823,302	64,353,128	5,791,781,520	97.20%	49.45%
	Asib6Ms	72,246,744	64,729,436	5,825,649,240	97.92%	48.86%
Females of stage II (>4 years old)	Asib1Mh-F	74,665,948	68,391,072	6,155,196,480	98.03%	45.91%
	Asib1Mp-F	73,798,512	66,751,484	6,007,633,560	97.34%	48.00%
	Asib1Ms-F	71,299,822	65,498,596	5,894,873,640	97.48%	50.08%
	Asib2Mh-F	83,710,718	77,999,142	7,799,914,200	99.11%	46.16%
	Asib2Mp-F	83,710,096	79,028,428	7,902,842,800	99.16%	47.22%
	Asib2Ms-F	81,447,328	77,768,116	7,776,811,600	99.22%	50.92%
	Asib3Mh-F	72,980,510	69,235,160	6,923,516,000	99.02%	47.17%
	Asib3Mp-F	60,687,670	55,755,734	5,575,573,400	98.91%	47.72%
	Asib3Ms-F	56,086,878	52,698,650	5,269,865,000	99.00%	49.66%
	Total	2,848,107,868	2,588,182,624	237,061,288,460

**Fig. 2:**
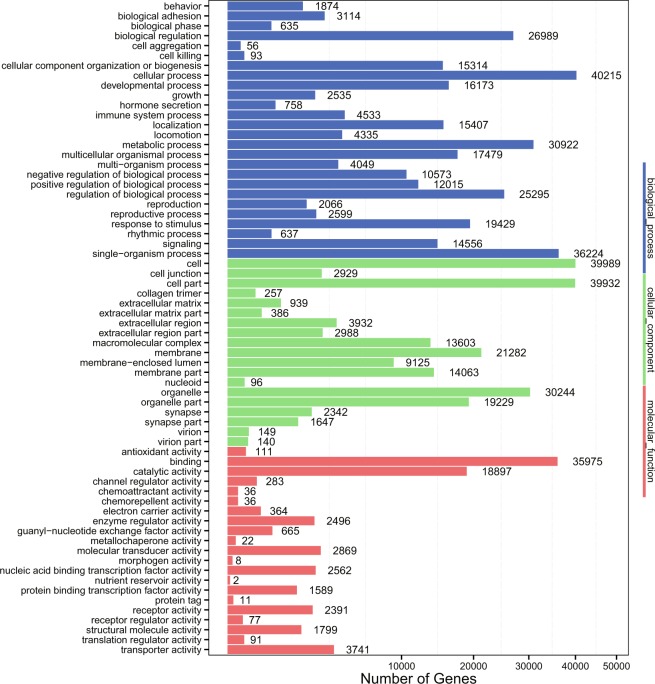
Gene Ontology classification of the *Acipenser sinensis* transcriptome. A total of 49,015 unigenes with BLASTx matched against the animal NR database were classified into three main GO categories (biological process, cellular component, molecular function) and 58 sub-categories. The left-hand scale on the y-axis shows the detailed names of the sub-categories. The x-axis indicates the number of unigenes in the same category.

A total of 74.5% of unigenes (90,853) were aligned to 7 databases (NR, Swiss-Prot, KEGG, COG, InterPro, GO and NT). A total of 76,268 unigenes were annotated to NR, 78,088 to NT, 67,964 to Swiss-Prot, 63,920 to KEGG, 30,194 to COG, 59,417 to InterPro and 53,038 to the GO database (Fig. 1b). For the functional annotation and classification analyses, with the annotation data based on the classification by GO, the cellular process, the cell and cell part category were the most frequent GO classification groups (Fig. 2). In COG analysis, 30,194 unigenes were annotated and classified into 25 functional categories (Supplementary Fig. 1). The largest cluster was “the general function prediction only”, followed by “Cell wall/membrane/envelope biogenesis” and “Signal transduction mechanisms”. Signal transduction; Cancers: Overview, Global and overview maps; Endocrine system; and Immune system were the top five annotated pathways according to KEGG classification analysis (Supplementary Fig. 2).

In BUSCO annotation analysis, the total number of Actinopterygii genes for evaluation was 4,584, and 85.7% of the total BUSCOs identified. In this evaluation data, the ‘Complete and single-copy BUSCOs’ was 42.5%, and the ‘Complete and duplicated BUSCOs’ was 43.2%.

**Fig. 3:**
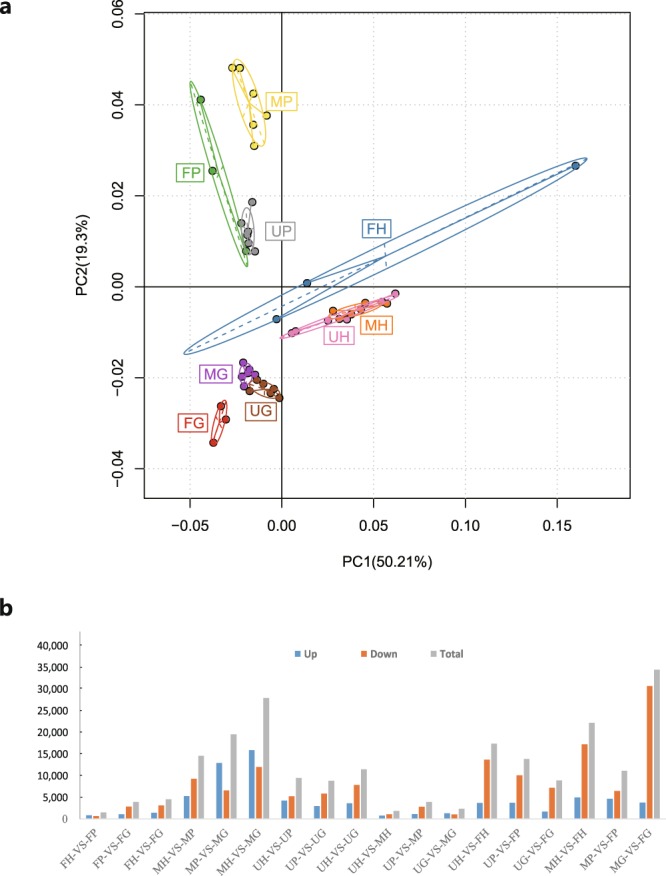
Principle component analysis (PCA) and different gene expression analysis in the *Acipenser sinensis* transcriptome. (**a**) PCA plot of 45 samples based on estimated expression values. (**b**) The statistics of different expression genes in different pairwise comparisons. A total of 18 pairwise comparisons among the HPG of different sexes were performed. (F: Female; M: Male; U: unknown; H: hypothalamus; P: pituitary; G: gonad).

Based on the gene expression data, PCA was performed for dimension reduction to extract a small number of representative features that can represent the effects of all genes. Herein, the PCA-based method on these data set succeeded in discriminating hypothalamus (H), pituitary (P) and gonad (G) from each other (Fig. 3a). However, the female sample displayed significant differences among replicates, especially for the female hypothalamus, possibly because the female samples came from different families. Then, Noiseq was used to calculate differential expression of two kinds of comparison purposes (18 pairwise comparisons including 9 in HPG and 9 in different sexes). Approximately 74.5–85.6% of the short reads for each individual were mapped to the final unigenes, indicating the good quality of the assembly results. In the female hypothalamus versus female pituitary (FH-VS-FP), 865 genes were upregulated, while 666 genes were downregulated. In the female pituitary versus female gonad (FP-VS-FG), 1,091 genes were upregulated, while 2,857 genes were downregulated. In the female hypothalamus versus female gonad (FH-VS-FG), 1,456 genes were upregulated, while 3,097 genes were downregulated. The number of DEGs greatly increased in the order of FH-VS-FP → FP-VS-FG → FH-VS-FG. A similar trend of gene expression in HPG also occurred in both males and individuals whose sexes could not be distinguished in stage I. Of the differentially expressed unigenes between males and females in the same kinds of tissues, 772 genes were upregulated, while 1,077 genes were downregulated in the unknown-sex hypothalamus versus male hypothalamus (UH-VS-MH); 3,696 genes were upregulated, while 13,685 genes were downregulated in the unknown-sex hypothalamus versus female hypothalamus (UH-VS-FH); and 4,981 genes were upregulated, while 17,211 genes were downregulated in the male hypothalamus versus female hypothalamus (MH-VS-FH). In addition to the comparisons above, the total results of pairwise comparisons of 9 kinds of tissues are described in Fig. 3b. To validate the repeatability and reproducibility of DEGs generated from annotated data, we re-amplified 10 sex-related genes by RT-PCR. Nine of the 10 genes had gene expression profiles similar to those from RNA-Seq, suggesting that the sex-related genes identified through RNA-Seq were highly accurate and reliable.

**Fig. 4:**
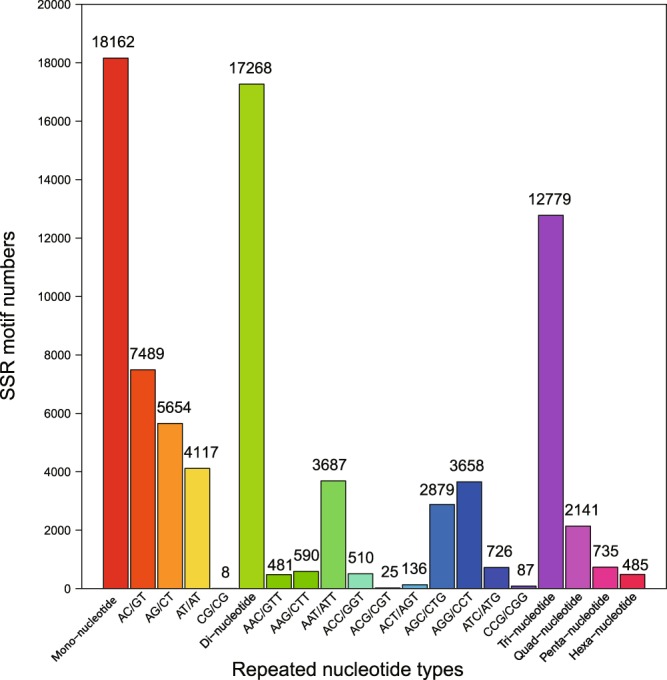
SSR distribution of *Acipenser sinensis* unigenes.

The unigene set generated a total of 51,570 SSRs, with the largest number of SSR motifs of mono-nucleotide repeats (18,162), followed by di-nucleotide repeats (17,628) and tri-nucleotide repeats (12,799) (Fig. 4).

## Usage Notes

For the first time, we reported the transcriptome resources of the integrated HPG of stage I and stage II in *A. sinensis*. The novel *de novo* assembly unigenes will provide the following: (i) abundant functional genes for further research on molecular evolution, genetic breeding and fish disease prevention; and (ii) an abundance of SSR markers in *A. sinensis*. In addition, the DEGs will offer possibilities (i) to understand the gene interaction and regulations of gonad development from stage I to II in *A. sinensis*, (ii) to explore the gene co-expression and sex differentiation in *A. sinensis*, and (iii) to reveal gene or tissue interaction models of the HPG axis in *A. sinensis*. These findings are also valuable for research on developmental regulation or gene expression patterns of tissue interaction, the identification of HPG- and sex-related genes, and developing SSR markers in other sturgeon species.

## Supplementary Information

### ISA-Tab metadata file

Download metadata file

### Supplementary information

Supplementary Figures

## Online-only Table

**Online-only Table 1:** Statistics of assembled unigenes from 45 transcriptome samples of *Acipenser sinensis.*

Sample Classification	Sample Name	Unigene Number	Unigene Length(nt)	Mean Unigene Length(nt)	N50	Distinct Clusters	Distinct Singletons	GC content(%)
Individuals of stage I (1 year old)	Asia1h	136,745	83,229,019	608	1,085	39,896	96,849	45.38
	Asia1p	133,508	78,671,828	589	1,007	37,769	95,739	45.69
	Asia1s	81,326	32,904,767	404	491	15,349	65,977	48.75
	Asia2h	143,263	89,375,925	623	1,125	42,909	100,354	45.43
	Asia2p	132,214	78,037,509	590	1,023	37,307	94,907	45.45
	Asia2s	121,534	69,354,029	570	941	33,834	87,700	46.20
	Asia3h	122,426	73,222,425	598	1,037	34,393	88,033	46.35
	Asia3p	129,274	76,029,334	588	1,029	36,145	93,129	45.72
	Asia3s	108,543	52,295,757	481	681	25,654	82,889	46.31
	Asia4h	126,556	67,751,855	535	839	33,536	93,020	46.16
	Asia4p	134,029	77,838,707	580	1,008	37,609	96,420	45.56
	Asia4s	106,304	60,670,762	570	957	27,805	78,499	47.20
	Asia5h	122,101	59,395,705	486	689	29,600	92,501	47.44
	Asia5p	139,379	79,806,132	572	964	38,975	100,404	46.11
	Asia5s	132,499	75,494,407	569	954	36,473	96,026	44.64
	Asia6h	115,753	60,236,671	520	776	30,336	85,417	47.62
	Asia6p	155,858	94,884,467	608	1,087	45,933	109,925	45.27
	Asia6s	120,288	57,366,733	476	687	29,322	90,966	44.22
Males of stage II (>4 years old)	Asib1Mh	125,451	86,424,239	688	1,336	38,263	87,188	46.01
	Asib1Mp	103,482	69,891,630	675	1,269	30,121	73,361	46.60
	Asib1Ms	126,458	91,587,291	724	1,440	41,788	84,670	46.31
	Asib2Mh	119,906	81,464,111	679	1,282	36,182	83,724	46.10
	Asib2Mp	114,620	76,486,712	667	1,259	33,847	80,773	46.57
	Asib2Ms	124,110	90,413,835	728	1,463	40,495	83,615	46.02
	Asib3Mh	108,294	73,758,866	681	1,270	32,739	75,555	46.40
	Asib3Mp	109,078	68,464,190	627	1,134	30,368	78,710	46.74
	Asib3Ms	115,414	82,355,316	713	1,412	36,095	79,319	46.33
	Asib4Mh	115,633	80,399,534	695	1,365	35,297	80,336	46.47
	Asib4Mp	126,747	80,469,294	634	1,175	36,323	90,424	46.45
	Asib4Ms	126,158	87,372,982	692	1,360	39,597	86,561	46.08
	Asib5Mh	121,304	80,350,698	662	1,259	35,634	85,670	46.41
	Asib5Mp	122,395	77,735,980	635	1,177	34,869	87,526	46.63
	Asib5Ms	124,621	88,175,276	707	1,398	40,053	84,568	46.51
	Asib6Mh	125,914	82,603,379	656	1,244	37,907	88,007	46.63
	Asib6Mp	106,979	67,431,297	630	1,139	29,526	77,453	46.85
	Asib6Ms	116,404	77,236,490	663	1,248	35,490	80,914	47.22
Females of stage II (>4 years old)	Asib1Mh-F	107,096	59,540,169	555	935	26,392	80,704	45.59
	Asib1Mp-F	110,039	63,291,880	575	1,008	27,842	82,197	46.50
	Asib1Ms-F	60,853	43,499,127	714	1,358	16,067	44,786	47.34
	Asib2Mh-F	85,890	69,161,046	805	1,387	28,595	57,295	43.91
	Asib2Mp-F	77,254	65,923,572	853	1,512	25,726	51,528	43.89
	Asib2Ms-F	40,374	37,323,089	924	1,638	12,166	28,208	46.37
	Asib3Mh-F	74,902	63,216,845	843	1,451	24,559	50,343	43.93
	Asib3Mp-F	72,157	56,965,898	789	1,317	22,470	49,687	44.04
	Asib3Ms-F	50,482	43,671,693	865	1,538	14,777	35,705	45.27
	Final unique gene	121,952	168,896,278	1,384	2,208	64,643	57,309	46.23
