# Supplementary material for: Hypothalamus-pituitary-gonad axis transcriptome profiling for sex differentiation in Acipenser sinensis
Source: Sci Data. 2019 Jun 13;6:87. doi: 10.1038/s41597-019-0099-1 (PMC6565624; doi:10.1038/s41597-019-0099-1)
Supplement: Supplementary file 2 — Supplementary Figures [file 41597_2019_99_MOESM2_ESM.pdf]

Hypothalamus-pituitary-gonad axis transcriptome profiling for sex differentiation in *Acipenser sinensis*

Contents:

Page numbers	File names	Descriptions
Page 2	Supplementary Figure 1	**COG functional classification of *Acipenser sinensis* unigenes.** A total of 30,194 unigenes were assigned to one or more of the 25 COG classification categories.
Page 3	Supplementary Figure 2	**KEGG pathways of *Acipenser sinensis* unigenes.** A total of 63,920 unigenes were assigned to one or more of the 33 KEGG classification categories.

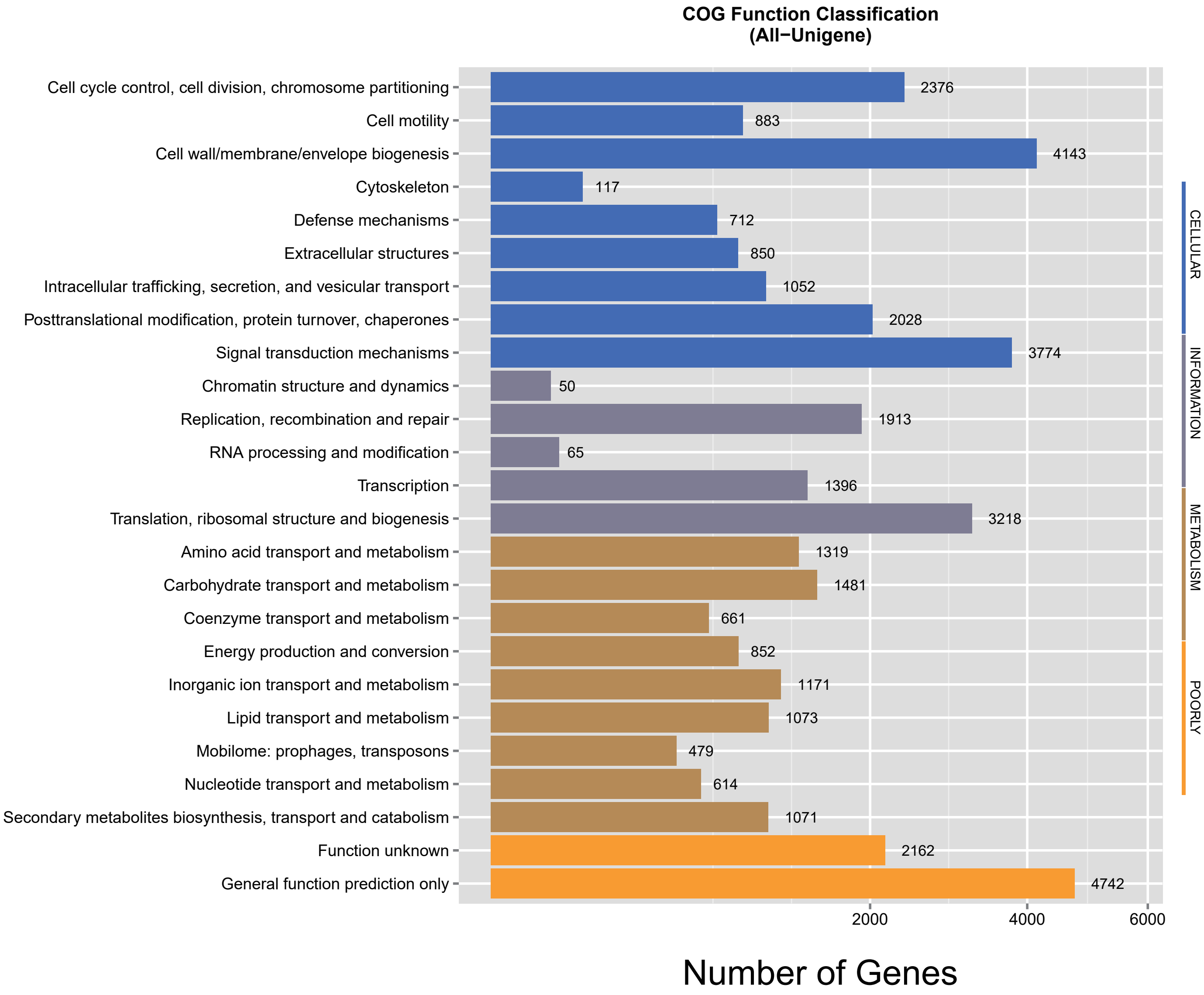

Supplementary Figure 1. COG functional classification of *Acipenser sinensis* unigenes. A total of 30,194 unigenes were assigned to one or more of the 25 COG classification categories.

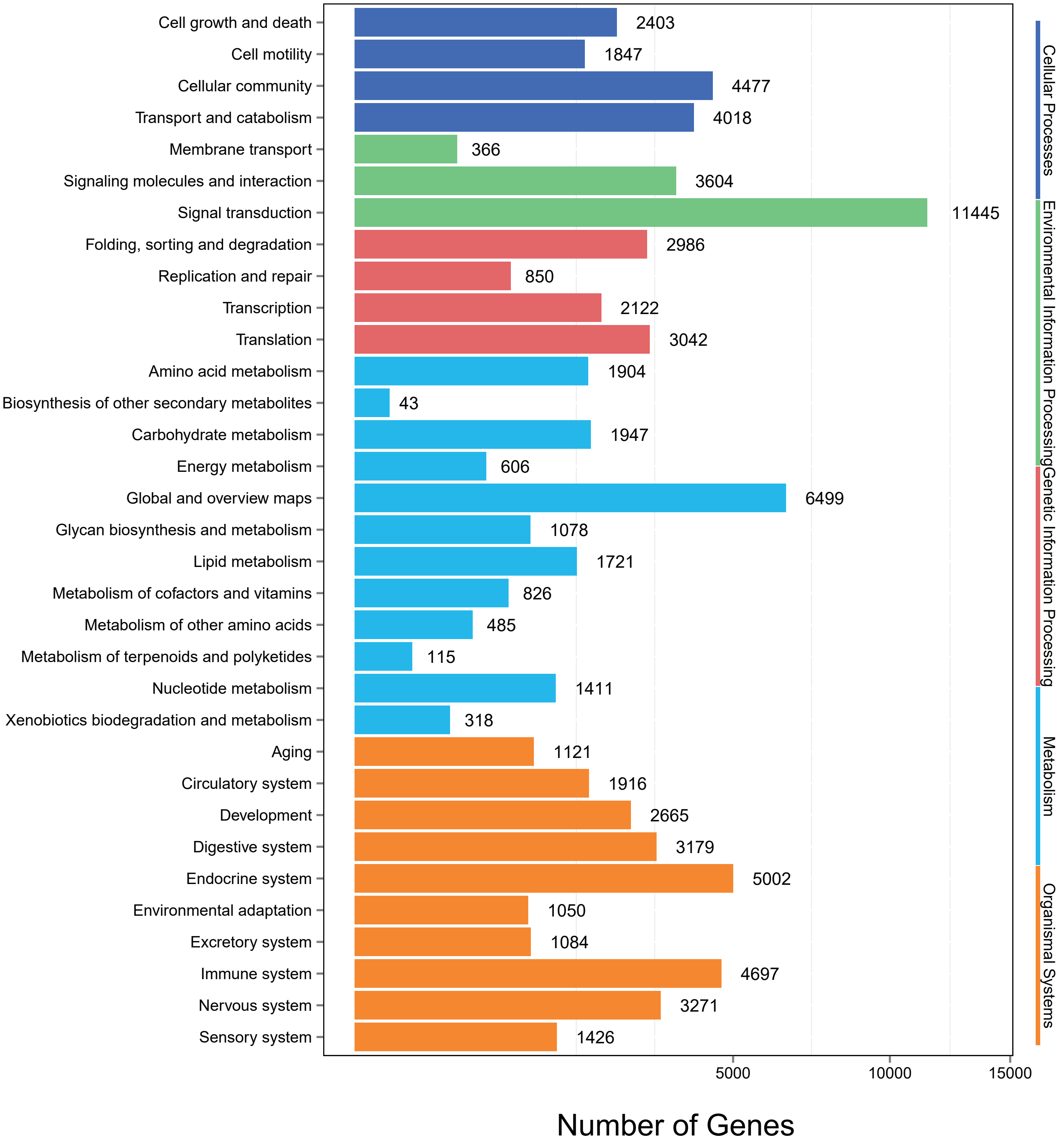

Supplementary Figure 2. KEGG pathways of *Acipenser sinensis* unigenes. A total of 63,920 unigenes were assigned to one or more of the 33 KEGG classification categories.
